# Data-balanced transformer for accelerated ionizable lipid nanoparticles screening in mRNA delivery

**DOI:** 10.1093/bib/bbae186

**Published:** 2024-04-25

**Authors:** Kun Wu, Xiulong Yang, Zixu Wang, Na Li, Jialu Zhang, Lizhuang Liu

**Affiliations:** Shanghai Advanced Research Institute, Chinese Academy of Sciences, Shanghai 201210, China; University of Chinese Academy of Sciences, Beijing 100049, China; Shanghai Advanced Research Institute, Chinese Academy of Sciences, Shanghai 201210, China; University of Chinese Academy of Sciences, Beijing 100049, China; Department of Computer Science, University of Tsukuba, Tsukuba 3058577, Japan; National Facility for Protein Science in Shanghai, Zhangjiang Laboratory, Shanghai Advanced Research Institute, Chinese Academy of Sciences; Shanghai Advanced Research Institute, Chinese Academy of Sciences, Shanghai 201210, China; University of Chinese Academy of Sciences, Beijing 100049, China; Shanghai Advanced Research Institute, Chinese Academy of Sciences, Shanghai 201210, China; University of Chinese Academy of Sciences, Beijing 100049, China

**Keywords:** ionizable lipid nanoparticles, data imbalance, transfection cliffs

## Abstract

Despite the widespread use of ionizable lipid nanoparticles (LNPs) in clinical applications for messenger RNA (mRNA) delivery, the mRNA drug delivery system faces an efficient challenge in the screening of LNPs. Traditional screening methods often require a substantial amount of experimental time and incur high research and development costs. To accelerate the early development stage of LNPs, we propose TransLNP, a transformer-based transfection prediction model designed to aid in the selection of LNPs for mRNA drug delivery systems. TransLNP uses two types of molecular information to perceive the relationship between structure and transfection efficiency: coarse-grained atomic sequence information and fine-grained atomic spatial relationship information. Due to the scarcity of existing LNPs experimental data, we find that pretraining the molecular model is crucial for better understanding the task of predicting LNPs properties, which is achieved through reconstructing atomic 3D coordinates and masking atom predictions. In addition, the issue of data imbalance is particularly prominent in the real-world exploration of LNPs. We introduce the BalMol block to solve this problem by smoothing the distribution of labels and molecular features. Our approach outperforms state-of-the-art works in transfection property prediction under both random and scaffold data splitting. Additionally, we establish a relationship between molecular structural similarity and transfection differences, selecting 4267 pairs of molecular transfection cliffs, which are pairs of molecules that exhibit high structural similarity but significant differences in transfection efficiency, thereby revealing the primary source of prediction errors. The code, model and data are made publicly available at https://github.com/wklix/TransLNP.

## INTRODUCTION

Despite the notable progress of messenger RNA (mRNA) drugs in protein replacement, cancer immunotherapy, gene editing, vaccines and more, the intrinsic instability and susceptibility to nucleases necessitate the development of an effective delivery system. Ionizable lipid nanoparticles (LNPs) are widely recognized as the preferred carriers for the effective delivery of mRNA into cells [1, 2]. LNPs are employed as mRNA drug delivery systems in the design of vaccines (for example, two COVID-19 mRNA vaccines [3, 4], mRNA-1273 and BNT162b2), targeting the prevention and treatment of viral infections (for example, human immunodeficiency virus [5], Powassan virus [6], Venezuelan equine encephalitis virus [7], dengue virus [8] and Ebola virus [9]), blood disorders (for example, hemophilia A [10], hemophilia B [11] and thrombotic thrombocytopenic purpura [12]), central nervous system diseases (for example, Friedreich’s ataxia [13]), skin conditions (for example, elastin deficiency syndrome [14]) and hearing loss [15], spanning a variety of disease areas. LNPs are typically composed of ionizable lipid, phospholipid, cholesterol and PEGylated lipid [16]. Notably, ionizable lipids constitute the highest molar ratio [17], forming the core structure of LNPs. Their primary functions include encapsulating mRNA within LNPs, facilitating its entry into the cytoplasm of target cells for ribosome binding and subsequent protein expression [18]. Research indicates that the molecular structure of ionizable lipids determines the formulation’s delivery efficiency and stability, as well as the metabolic kinetics and toxicity of LNPs [19]. Therefore, the screening of efficient novel ionizable lipids is pivotal for the rapid advancement of nucleic acid technologies.

The task of screening ionizable lipids is complex and costly, requiring the construction of an extensive lipid library to test hundreds of thousands of compounds for suitable lipids for mRNA transfection into different cells. In this context, the multi-component reaction method has become a commonly used approach for the design and screening of ionizable lipids. For example, a three-component reaction mediated by isocyanide was employed to synthesize a pilot library comprising 1080 lipids [20]. In addition, a three-component reaction system was employed to synthesize and evaluate a library of 720 biodegradable ionizable lipids [21]. Although progress has been made in the screening of ionizable lipids using three-component reactions, the synthetic process of three-component reactions is relatively complex, involving multiple steps, which may lead to reduced yields. In contrast, lightGBM [22] is a machine learning method which introduced a prediction model based on 325 data samples of mRNA vaccine LNPs formulations to forecast the transfection efficiency of LNPs. Due to the limited number of samples in this machine learning method like LightGBM, it neglects the feature learning capability for small samples and demonstrates poor generalization. However, deep learning methods can learn patterns and correlations from large amounts of data, offering greater flexibility in capturing complex structure–property relationships while avoiding some of the experimental challenges in synthetic chemistry and addressing the shortcomings of machine learning generalization ability. AGILE [23] introduced a prediction model for LNPs transfection efficiency based on GIN graph convolutional networks. Although AGILE tackles the challenge of limited data through pretraining and fine-tuning, it neglects the issue of imbalanced data, which led to a big forecast error.

AGILE and lightGBM have shown that the challenge in predicting LNPs transfection efficiency lies in addressing the issues of limited [24–26]and imbalanced sample data [27–30]. Inspired by AGILE, we find that pre-training enhances the model’s ability to learn molecular structures, addressing the scarcity of LNPs data. Consequently, we propose TransLNP, a Transformer-based model for predicting LNPs transfection efficiency. Molecular representation is crucial in predicting molecular properties [31–34]. In comparison with AGILE’s pre-training, TransLNP fully leverages both coarse-grained atomic sequence information and fine-grained atomic spatial relationship information to perceive the connection between molecular structure and transfection efficiency. Additionally, in response to the imbalance issue caused by limited LNPs data, we introduce the BalMol bolck, which balances the data by smoothing label distributions and molecular feature distributions. The combination of TransLNP and the BalMol bolck reduces the mean squared error (MSE) to 1.47 on the AGILE dataset compared with AGILE. Additionally, when compared with decision trees, support vector regression (SVR) and graph convolutional neural network algorithms, it demonstrates state-of-the-art performance. Despite achieving good results, we are still committed to identifying the root causes of prediction errors. In this process, we have discovered the phenomenon of molecular transfection cliffs. Molecular transfection cliffs indicate that minor changes in molecular structure can lead to significant differences in transfection efficiency, which is the primary source of prediction errors.

In this study, our primary contributions can be summarized as follows:

1. In the field of LNPs, we introduce TransLNP, a model based on Transformer. The TransLNP model employs a pretraining and fine-tuning strategy for the screening of ionizable lipids, making full use of both coarse-grained atomic sequence information and fine-grained atomic spatial relationships to perceive the connection between molecular structure and transfection efficiency.
2. We introduce the BalMol bolck, designed to address the issue of imbalanced datasets by balancing the similarity between neighboring targets. The balance of transfection efficiency labels is primarily achieved through kernel functions. Balancing molecular features primarily involves smoothing the variance and mean of the molecular features, and ultimately updating the molecular features through the CORrelation ALignment method.
3. Through our analysis we found that the main source of error is the phenomenon of molecular transfection cliffs. Highly similar pairs of molecules are screened through calculations of substructure similarity, scaffold similarity and SMILES string similarity. By comparing the transfection efficiency of highly similar pairs, molecular transfection cliffs are observed that significant differences in transfection efficiency exist, despite the structural similarity.
4. Extensive model comparisons and ablation experiments have been conducted to demonstrate the effectiveness of the proposed model. Our approach exhibits superior performance compared with state-of-the-art deep learning models.

## MATERIALS AND METHODS

**Figure 1:**
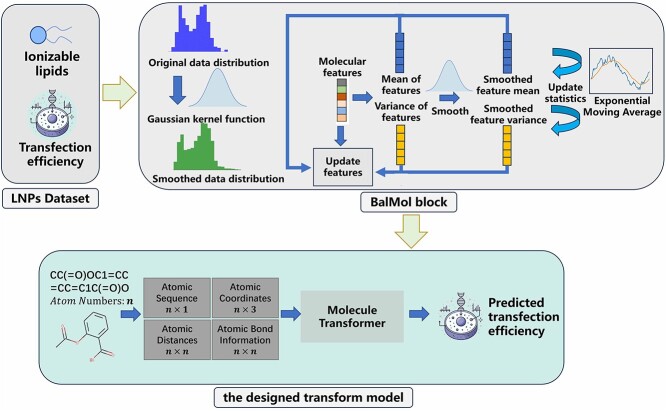
The overall architecture for predicting the properties of ionizable lipids begins with obtaining transfection efficiency datasets for various ionizable lipids. Subsequently, the BalMol block is used to smooth the distribution of labels and molecular features for balancing the data of LNPs. Finally, the TransLNP model is employed to predict transfection efficiency.

The overall architecture of TransLNP for predicting the properties of ionizable lipid is illustrated in Figure 1. TransLNP encompasses the LNPs dataset, BalMol block and the designed transfomer model. Firstly, the dataset consists of ionizable lipids and their transfection efficiency data. Furthermore, the transfection efficiency is subjected to data balancing within the label smoothing section of the BalMol block. Subsequently, TransLNP extracts features of ionizable lipids, which are then subjected to data balancing within the feature smoothing section of the BalMol block. Ultimately, TransLNP captures the relationship between molecular structure and transfection efficiency to achieve prediction.

### Model Design

**Figure 2:**
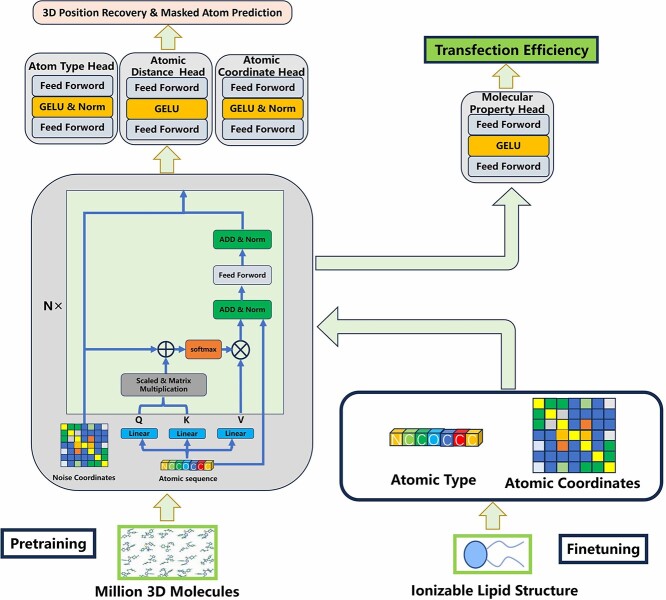
The design architecture of the TransLNP model, which consists of two main components. **Left**: Pretraining, where molecular structure is learned through atom masking, predicting atom types, coordinates and interatomic distances. **Right**: Fine-tuning, predicting transfection efficiency using labeled data.

Graph neural networks (GNNs) [35] and transformers [36, 37] are commonly used backbone networks in the field of molecular research. We opted for the Transformer architecture due to GNN’s limitations in capturing long-range atom interactions [38–40], while Transformers facilitate unconstrained interactions among all graph nodes, irrespective of local structures [41–43]. Inspired by the Unimol method [44], we design the TransLNP model shown in Figure 2.

Firstly, the molecular formula of ionizable lipids is represented as an atomic sequence, atomic distances, atomic coordinates and atomic edge types. When performing masked language modeling tasks, 15$\%$ of the atoms are randomly masked and mask tokens are introduced. Subsequently, an embedding layer is then employed to map the atomic sequence into a representation of atomic types, imbuing each atom with semantic information. For addressing interactions between atoms, a Transformer self-attention module is utilized. Masked atomic types are linearly represented as queries (Q), keys (K) and values (V) in the self-attention module. The attention bias in the self-attention mechanism is based on the representation of atomic distances and edge types. Equation 1 describes the interaction of atomic types and attention bias. $\sqrt{d}$ represents the dimensions of vectors Q, K and V. 


(1) \begin{align*}& attention = \text{softmax}\left(\frac{QK}{\sqrt{d}} + bias\right)V\end{align*}

After considering the relationships between atoms, uniformly distributed noise was introduced and added to the 3D coordinates of the molecules. The coordinate updating process effectively incorporated noise into the molecular representation. Equation 2 represents the coordinate updating process. $ Ac $ represents the 3D coordinates of the molecule, $ Ac^{T} $ represents the transpose of the matrix $ Ac $, $ \text{atom{\_}num} $ is the number of atoms, $ \text{attn{\_}probs} $ denotes the attention distribution from atom pairs to coordinates and $ \odot $ signifies element-wise multiplication. 


(2) \begin{align*}& \text{update{\_}coord} = \text{Ac} + \frac{\text{Ac} \otimes \text{Ac}^{\top} - \text{Ac}^{\top} \otimes \text{Ac}}{\text{atom{\_}num}} \odot \text{attn{\_}probs}\end{align*}

Finally, different heads (atomic type head, atomic coordinate head and Euclidean distance head between atoms) are employed for multi-task learning in the pre-trained model. These heads are used for predicting the Euclidean distances between atoms, masked atomic coordinates and atomic types, respectively. We utilize Smooth L1 as the loss function for predicting the Euclidean distances and masked atomic coordinates, while cross-entropy is employed as the loss function for predicting atomic types.

For the fine-tuning phase, additional information is extracted from the limited labeled data. Specifically, the pre-trained model is initialized and fine-tuned with labeled LNPs formulation data. This process involves converting molecular structures into atom types, atomic coordinates, interatomic distances and bond information, following the data processing procedure from pre-training. Finally, property predictions are made using the molecular prediction head.

### Data balancing processing

To handle imbalanced label data, inspired by label distribution smoothing (LDS) and feature distribution smoothing (FDS) methods [28], we primarily focus on understanding the characteristics of ionizable lipid molecules and the distribution of transfection efficiency labels [28] shown in the BalMol block part of Figure 1.

#### Label balancing processing

The core idea for balancing transfection efficiency label data lies in leveraging the similarity between adjacent targets through explicit distribution smoothing in both label and feature spaces using a kernel. Firstly, kernel density estimation is performed on the dataset labels to understand the potential imbalance in a continuous target context. Next, performing one-dimensional convolution with the symmetric kernel function on labels achieves a smoothing effect, as shown in Eq. 3. Here, $p(y)$ represents the frequency of occurrence of label $y$ in the training data, and $p(y^{\prime})$ represents the smoothed frequency of label $y^{\prime}$ after convolution. After obtaining $p(y^{\prime})$, the loss function is reweighted by multiplying it by the reciprocal of $p(y^{\prime})$. The reciprocal operation is performed to assign greater weights to categories with fewer instances. As the reciprocal is larger, it results in a higher weight in the loss function. This reweighting helps the model focus more on categories that are relatively less frequent in the training data, thereby enhancing the model’s adaptability to the overall data distribution. 


(3) \begin{align*}& \label{eq7}p(y^{\prime}) \triangleq \int\limits_{y}{k(y,y^{\prime})p(y)dy}\end{align*}

#### Molecular Feature Balancing

The phenomenon of data imbalance exists not only in the labels of data but also in molecular features. Balance molecular features are similar to balancing data labels. Kernel function smoothing is applied to the variance and mean of features. Simultaneously, the mean and variance are updated using the exponential moving average (EMA) method, and finally, molecular features are updated using the CORrelation ALignment method [45].

Firstly, similar to the treatment of label means, labels are divided into several intervals. The number of samples in the $i$-th interval is denoted as $N_{i}$, and the $j$-th molecular feature in the $i$-th interval is denoted as $F_{j}$. Equations 4 and 5 represent the calculation methods for the mean and variance of molecular features. 


(4) \begin{align*} \label{eq8} \bar{F} &= \frac{1}{N_{i}} \sum_{j=1}^{N} F_{j},\end{align*}

(5) \begin{align*} \label{eq9}\sigma_{F} &= \frac{1}{N_{i}} \sum_{j=1}^{N} (F_{j} - \bar{F})^{2}.\end{align*}

After obtaining the mean and variance of molecular features, following the approach outlined in Eq. 3, symmetric kernel functions are convolved independently with the mean and variance through one-dimensional convolution to obtain $\bar{F^{\prime}}$ and $\sigma _{F^{\prime}}$. To enhance the accuracy of property prediction and increase the robustness of the model, we introduce EMA and momentum for updating the mean and variance. Specifically, the updating process of the mean and variance adopts the form of EMA shown in Eq. 6, where the incorporation of momentum enhances the memory of past information, aiding in robust adaptation to dynamically changing data distributions. $S_{t}$ is the EMA value for the current epoch, $Y_{t}$ represents the mean and variance for the current epoch and $\alpha $ is the smoothing factor associated with momentum. 


(6) \begin{align*}\label{eq10}& S_{t} = \alpha \cdot X_{t} + (1 - \alpha) \cdot S_{t-1}.\end{align*}

Finally, based on $\bar{F^{\prime}}$ and $\sigma _{F^{\prime}}$, the molecular feature $F$ is updated using the CORrelation ALignment method. CORrelation ALignment is an unsupervised domain adaptation technique based on standard whitening and recoloring. Equation 7 illustrates the process of standard whitening and recoloring, where the role of $[a, b]$ is to constrain the values of $\frac{\sigma _{F^{\prime}}}{\sigma _{F}}$ within this interval. Finally, Algorithm 1 describes the overall process of the data-balanced transformer for LNPs transfection efficiency prediction. 


(7) \begin{align*}\label{eq11}& F_{update} =\min\left(\max\left(\sqrt{\frac{\sigma_{F^{\prime}}}{\sigma_{F}}},a\right),b\right)(F-\bar{F})+\bar{F^{\prime}}.\end{align*}

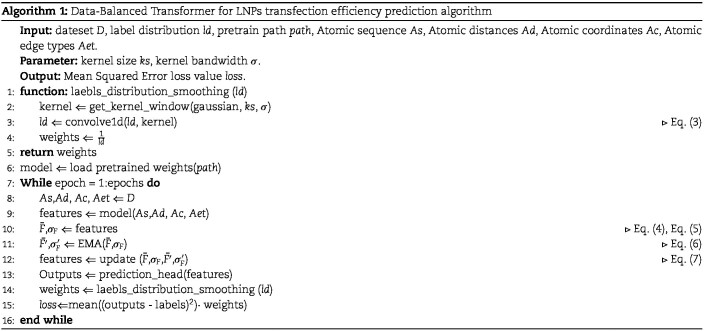


### Dataset description

The dataset includes pre-training and fine-tuning datasets. Firstly, the pre-training dataset is derived from previous work [44], consisting of 19 datasets.(The 19 datasets include Targetmol (link), Chemdiv (link), Enamine (link), Chembridge (link), Life Chemical (link), Specs (link), Vitas-M (link), InterBioScreen (link), Maybridge (link), Bionet-Key Organics (link), Asinex (link), UkrOrgSynthesis (link), Eximed (link), HTS Biochemie Innovationen (link), Princeton BioMolecular (link), Otava (link), Alinda Chemical (link), Analyticon (link), ZINC (link) and ChemBL (link).) The dataset comprises 19 million molecules, with each molecule randomly generating 11 conformations, resulting in a total of 209 million conformations. The fine-tuning dataset provided by AGILE method encompasses 1200 ionizable lipid structures represented in SMILES molecular notation, along with corresponding label data denoting transfection efficiency in Hela cells(https://github.com/bowang-lab/AGILE). The synthesis process of the dataset commences with the high-throughput synthesis platform based on the Ugi 3CR. This platform facilitates the synthesis of 1200 ionizable lipids, comprising 20 diverse head groups, 12 carbon chains and 5 carbon chains with isocyanide head groups. Following the synthesis, LNPs are produced using four classical formulations incorporating ionizable lipids, helper lipid, cholesterol and polyethylene glycol-phospholipid conjugate. Ultimately, the transfection efficiency of mRNA in Hela cells is assessed by encapsulating firefly luciferase (Fluc) mRNA within LNPs, allowing for the measurement of Fluc protein expression activity.

### Experimental processing

For model pretraining, the prediction of atomic types utilizes the cross-entropy loss function with a weight of 1. For the prediction tasks related to atomic coordinates and interatomic distances, the Smooth L1 loss function is chosen with weights set at 5 and 10, respectively. In terms of the network architecture, the number of layers (N) is set to 5, and the batch size is set to 128, as illustrated in Figure 2. During the training process, a linear decay strategy is employed for the learning rate, and the optimizer selected is Adam with epsilon set to 1e-6. The decay rate for the first momentum is set to 0.9, while the decay rate for the second momentum is set to 0.99.

For model fine-tuning, during the training process, the hyperparameters include a learning rate of 5e-5, a batch size set to 4, and data partitioning methods encompass both random and scaffold. The number of epochs is set to 200, with a warm-up ratio of 0.1. Regarding balancing, parameters for label data balancing consist of setting the number of bins to 20, defining a data range of (-2, 16) and opting for the Gaussian kernel function as the symmetric kernel function, with kernel size and sigma set to 5 and 2, respectively. For feature balancing, parameters include setting the number of bins to 50, defining a data range of (-2, 4) and choosing the Gaussian kernel function as the symmetric kernel function, with kernel size and sigma set to 15 and 2, respectively. Momentum is set to 0.9.

#### Baselines

In this section, we compare our method with five previous approaches, including self-supervised graph neural networks, graph diffusion based on hierarchical molecular syntax, random forest, lightGBM and SVR methods. These comparisons aim to assess the superiority of our proposed method in terms of performance and validate its effectiveness from multiple perspectives.

1. **AGILE:** A method based on graph convolutional neural networks uses contrastive learning for pre-training. It pre-trains on a dataset comprising 60 000 lipid structures and fine-tunes on 1200 high-throughput synthesized ionizable lipids. The final selection process involves choosing the top 15 ionizable lipids exhibiting high transfection efficiency.
2. **Random Forest:** It is a method that integrates multiple decision trees for molecular property prediction. Initially, the SMILES format of the data is transformed into molecular fingerprint features. Each decision tree is trained on randomly selected feature subsets and different subsets of the training data. The prediction of molecular properties is achieved through the ensemble of multiple models.
3. **SVR:** The method adopted is based on support vector machines. SVR utilizes support vectors to define the optimal hyperplane, capturing the relationship between features and property values. By selecting different kernel functions, such as RBF, linear and polynomial kernels, SVR can map the input space to a mathematical function in a high-dimensional feature space.
4. **Geo-DEG:** It is a methodology based on learnable molecular grammar, geometric structures in molecular graphs and data-efficient property prediction achieved through graph neural diffusion [46]. By employing a learnable hierarchical molecular grammar, the model is capable of generating molecular structures based on acquired production rules. Molecular property prediction is then performed through graph neural diffusion on the induced geometry.
5. **lightGBM:** It is a method based on gradient boosting decision trees. It incrementally enhances model performance by constructing leaf nodes of decision trees, utilizing an algorithm based on histograms to accelerate the selection of split points. In the prediction of molecular properties, molecular fingerprint features can be extracted and LightGBM parameters adjusted to optimize model performance.

## RESULT

### Performance comparison with other methods

The 10-fold cross-validation performances of our method are shown in Table 1 for the transfection efficiency dataset. Each method employed both random and scaffold data splitting approaches. Random splitting ensures a good mix of data in both sets, which helps the model generalize better to unseen data. However, if the training and test sets contain many similar molecules, this could make the prediction task relatively easy. Compared with random splitting, scaffold-based splitting is more challenging, as the sets of molecular scaffolds in different subsets do not overlap, requiring the model to have better generalization capabilities across different molecular scaffolds, more accurately reflecting real-world scenarios [47].

**Table 1:** Predictive results for the transfection efficiency dataset with MSE, MAE (mean absolute error), Pearson correlation coefficient (pearsonr), $R^{2}$ (the larger the value, the better). Each method is employed random and scaffold data splitting approaches, with the best result highlighted in bold. Ran-For stands for Random Forest

Splitting	Random						Scaffold					
Method	TransLNP	AGILE	Ran-For	SVR	GEO-DEG	lightGBM	TransLNP	AGILE	Ran-For	SVR	GEO-DEG	lightGBM
MSE	**5.17**	7.41	5.73	5.90	5.89	7.10	**4.91**	6.38	7.72	6.96	6.37	6.24
MAE	1.76	2.17	1.89	1.92	**1.74**	2.08	**1.85**	1.92	2.06	2.28	2.03	2.05
pearsonr	**0.69**	0.51	0.66	0.64	0.57	0.56	**0.58**	0.46	0.24	0.34	0.55	0.39
$R^{2}$	**0.48**	0.25	0.42	0.40	0.27	0.28	**0.31**	0.11	-0.08	0.03	0.24	0.13

**Figure 3:**
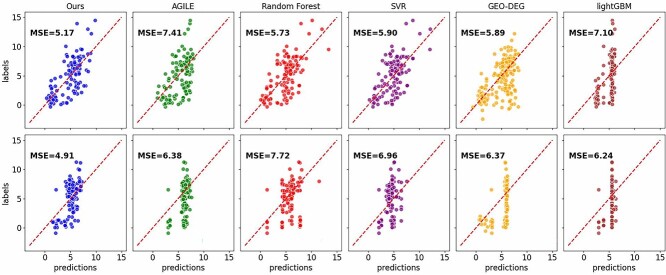
Scatter plots of predictions and labels for five methods. **The first row**: random splitting. **The second row**: scaffold splitting.

Under both splitting methods, our approach demonstrated the best performance in terms of MSE, mean absolute error (MAE), Pearson correlation coefficient and $R^{2}$. Our data originate from AGILE, but compared with our method, for example, in random splitting, MSE and MAE decreased by 2.24 and 0.41, respectively, and Pearson correlation coefficient and $R^{2}$ increased by 0.18 and 0.22, respectively. From the perspective of data splitting, the difference in results of our method under both types of data splitting was smaller than the other five methods under the two types of data splitting, indicating our method’s strong generalization capability. Figure 3 illustrates scatter plots of predictions and labels for the five methods under two different data splitting approaches. Compared with the other five methods, our approach demonstrates the highest degree of alignment between predictions and labels. The data points cluster closely around the ideal line, indicating superior prediction accuracy and stronger correlation compared with the other methods, especially under the more challenging scaffold data splitting. Furthermore, it is evident that the overall correlation for scaffold data splitting is lower than that for random splitting, emphasizing the challenging nature of scaffold data splitting.

### Ablation experiment

Data balancing in the BalMol block is achieved through LDS and molecular FDS. In LDS, we combine balancing methods with inverse weighting in the loss function. To validate the feasibility of the data balancing method and the superiority of inverse weighting, in this section, ablation experiments involve transfection efficiency predictions using LDS, FDS and LDS+FDS separately shown in Table 2, as well as transfection efficiency predictions using different weighting schemes shown in Table 3.

**Table 2:** The table for transfection efficiency predictions using three data balancing methods (LDS, FDS and LDS+FDS) and without using balancing method (None) under random and scaffold data splitting, with the best results highlighted in bold. For each data balancing method, both pre-training and non-pre-training approaches are employed. ‘pre’ denotes the usage of pre-training, while ‘no_pre’ indicates the absence of pre-training

Splitting	Random								Scaffold							
Method	LDS		FDS		LDS+FDS		None		LDS		FDS		LDS+FDS		None	
	pre	no_pre	pre	no_pre	pre	no_pre	pre	no_pre	pre	no_pre	pre	no_pre	pre	no_pre	pre	no_pre
MSE	**5.17**	9.31	5.57	9.41	5.55	9.64	5.54	9.56	5.39	7.32	**4.91**	7.18	5.10	7.38	5.13	7.21
MAE	**1.76**	2.54	1.79	2.57	1.85	2.61	1.81	2.58	1.91	2.31	**1.85**	2.28	1.87	2.33	1.88	2.30
pearsonr	**0.69**	0.27	0.67	0.27	0.67	0.21	0.67	0.22	0.52	0.08	**0.58**	0.18	0.55	0.08	0.56	0.12
$R^{2}$	**0.48**	0.06	0.44	0.05	0.44	0.02	0.44	0.03	0.25	-0.02	**0.31**	0.00	0.29	-0.03	0.28	0.00

**Table 3:** The table of different weighting schemes in LDS. The weighting schemes include logarithmic (log), exponential (exp), inverse square (InverSq), inverse square root (InverSqrt) and direct inverse (InvDirect)

	Log	Exp	InverSq	InverSqrt	InvDirect
MSE	5.57	5.53	6.10	5.38	**5.17**
MAE	1.79	1.77	1.91	0.76	**1.76**
pearsonr	0.67	0.67	0.63	0.68	**0.69**
$R^{2}$	0.44	0.44	0.38	0.45	**0.48**

Table 2 represents the results of transfection efficiency predictions using three data balancing methods (LDS, FDS and LDS+FDS) and without using any balancing method (None) under random and scaffold data splitting approaches. It is worth noting that the combination of LDS and FDS (LDS+FDS) does not yield the expected optimal performance, and it is even comparable with not using any data balancing method (None). This phenomenon partly arises from the issue of over-correction when both LDS and FDS are applied simultaneously, resulting in excessive data smoothing and the loss of some helpful information for prediction tasks. In pre-training, Atomic sequence, Atomic coordinates, Atomic distances and Atomic bond information are used as inputs to learn molecular structure prediction through masking atoms and 3D positions. To demonstrate the contribution of these types of information for improving model accuracy, molecular property prediction is conducted on the dataset with and without pre-training. As shown in Table 2, when using the same data balancing method, the results of prediction with pre-training significantly outperform those without pre-training.

**Figure 4:**
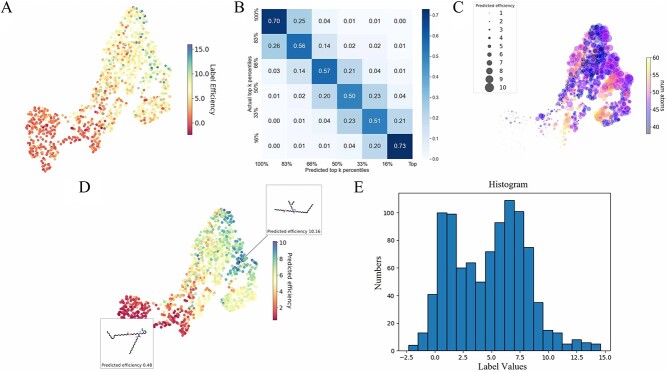
The label distribution and molecular feature plots under random splitting. (**A**) UMAP plot of labels and molecular features. (**B**) The confusion matrix computed on the predictions and labels. (**C**) The feature relationship plot between predictions and the number of atoms. (**D**) UMAP plot of predictions and molecular features. (**E**) The labels distribution plot.

**Figure 5:**
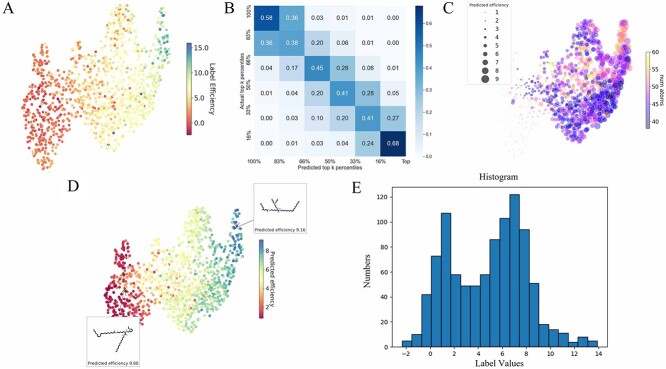
The label distribution and molecular feature plots under scaffold splitting. (**A**) UMAP plot of labels and molecular features. (**B**) The confusion matrix computed on the predictions and labels. (**C**) The feature relationship plot between predictions and the number of atoms. (**D**) UMAP plot of predictions and molecular features. (**E**) The labels distribution plot.

Figures 4 and 5 present the molecular feature maps under random and scaffold splitting. The UMAP plots and label histograms reveal an imbalance in both feature distribution and label distribution, underscoring the necessity of the BalMol block for achieving data balance. The features used as input for UMAP are derived from the encoding process by the transformer encoder, as illustrated in Figure 2, where they represent the features inputted into the Molecular Property head.

In the application of LDS, we achieve data balancing by taking the inverse of the smoothed label distribution and multiplying it with the loss function. To validate the effectiveness of the direct inverse approach, we compare it with several other weighting methods, including logarithmic weighting, exponential weighting, inverse square weighting and inverse square root weighting. Table 3 shows that the direct inverse weighting method achieved the best performance in terms of the four evaluation metrics.

### Molecular transfection cliffs

Observations from the UMAP plots in Figures 4 and 5 indicate points that are close in distance but have significant differences in color, which shows ionizable lipids with very similar structures might exhibit significantly different transfection efficiencies. To validate our hypothesis, we introduced the concept of transfection cliffs [48]. Transfection cliffs are defined and evaluated by comparing the differences in transfection of LNPs pairs against their structural similarities, which is instrumental in understanding the nonlinear relationship between the structure and function of ionizable lipids.

The key step in identifying transfection cliffs is the analysis of structural similarity. We calculate the structural similarity between molecular pairs using substructure similarity, scaffold similarity and SMILES string similarity. Substructure similarity is determined by computing the Tanimoto coefficient of Extended Connectivity Fingerprints for two molecules, reflecting the similarity in molecular substructures. Scaffold similarity involves calculating the Tanimoto coefficient of Molecular ACCess System keys for the atomic scaffolds of two molecules, representing the similarity in their molecular scaffolds or core structures. SMILES string similarity is assessed by computing the Levenshtein distance between the SMILES string representations of two molecules, indicating differences in atom types and bonding patterns.

Molecular pairs with an average structural similarity score above 0.9 are defined as highly similar. For these highly similar pairs, we calculate their biological transfection differences (TD) by subtracting the logarithm of their transfection efficiencies shown in Eq. 8. 


(8) \begin{align*}& TD(m_{1},m_{2}) = |\log_{10}(2^{y_{m_{2}}}) -\log_{10}(2^{y_{m_{1}}})|,\end{align*}

where $m1$ and $m2$ represent a pair of molecules, and $y_{m_{2}}$ and $y_{m_{1}}$ are the corresponding transfection efficiency of $m1$ and $m2$. Since the original values in the dataset have been processed using a logarithm with a base of 2, we compute the corresponding values with a base of 10. Pairs with a biological transfection difference of 10 times or greater (absolute value of the logarithmic subtraction greater than 1) are identified as transfection cliff pairs.

**Figure 6:**
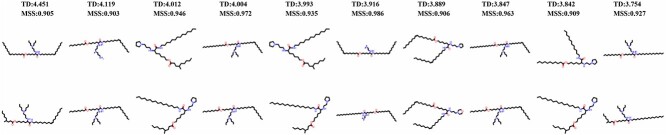
Top 10 molecular transfection cliff pairs with transfection differences. MSS represents molecular structural similarity (maximum value is 1), and TD represents the transfection difference.

**Figure 7:**
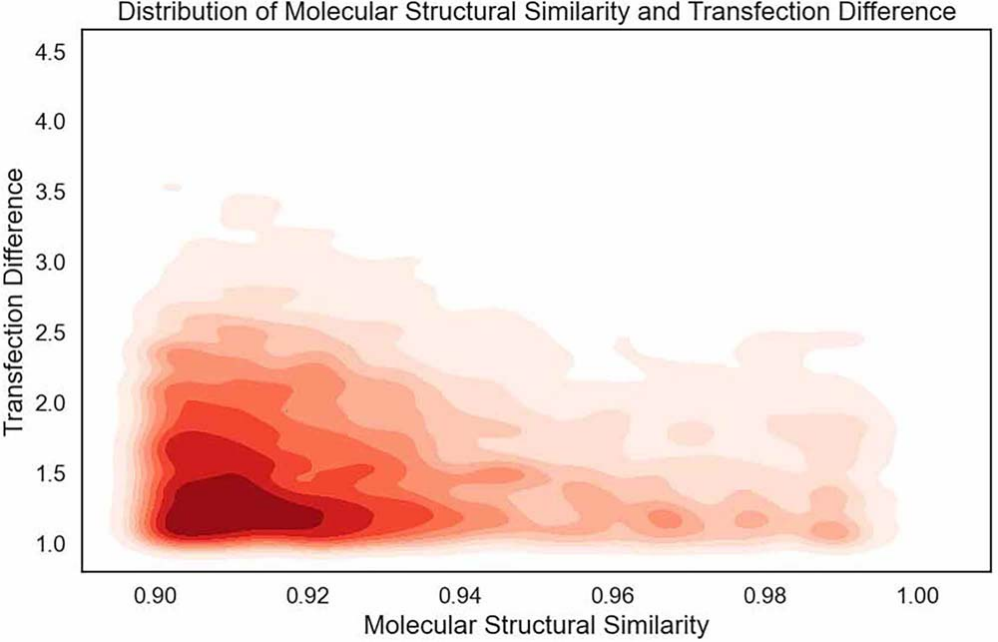
The heatmap of molecular structure similarity and transfection differences. The X-axis represents molecular structural similarity (with a maximum value of 1), and the Y-axis represents the transfection difference.

We obtain 4267 transfection cliff pairs from the LNPs dataset. Figure 6 presents the top 10 molecular transfection cliff pairs with the greatest TD, while Figure 7 illustrates the relationship between molecular structure similarity (MSS) and transfection difference (TD) among transfection cliff pairs. From the two images, we can observe that molecules with highly similar structures can differ by thousands to tens of thousands of times in transfection efficiency. Despite highly similar molecular structures, the significant difference in transfection efficiency makes it more challenging for neural networks to capture both molecular features and transfection efficiency, resulting in some level of prediction error.

## DISCUSSION AND CONCLUSION

In this work, we aim to achieve high-precision prediction of the transfection efficiency of LNPs in mRNA drug delivery systems. Due to the limitation of LNPs data, we propose TransLNP for predicting the properties of LNPs, adopting a learning strategy that combines pre-training and fine-tuning. Considering the imbalanced nature of LNPs dataset, we also present the BalMol block for data balance. Specifically, ionizable lipids are described in four aspects: atomic sequences, atomic coordinates, interatomic distances and bonds between atoms. Data balance is achieved through the smoothing of label distributions and molecular feature distributions. The training results demonstrate that our model exhibits competitive performance compared with the state-of-the-art deep learning models and machine learning models used in molecular property prediction. Additionally, we conduct ablation experiments to validate the critical role of label distribution smoothing and molecular feature distribution smoothing in enhancing the accuracy of transfection efficiency prediction. Through these experiments and analyses, the TransLNP model not only showcases an effective method for predicting LNPs properties but also provides valuable insights into the application of deep learning in the prediction of molecular properties.

We analyze the reasons for prediction errors by selecting transfection cliffs pairs of molecules, and discover that even though the molecular structures are highly similar, there can still be differences in transfection efficiency by orders of magnitude. This phenomenon makes it extremely challenging for the model to capture the relationship between molecular features and properties. Therefore, our future work will focus on improving our model specifically in response to the characteristics of molecular transfection cliffs.

**Key Points**

- We propose TransLNP based on Transformer to aid in the selection of LNPs for mRNA drug delivery systems, which can combine coarse-grained atomic sequence information and fine-grained atomic spatial relationship information to understand LNPs structure and transfection efficiency, with pretraining used to improve predictions due to limited experimental data.
- We propose the BalMol block to solve the issue of LNPs data imbalance by smoothing the distribution of labels and molecular features.
- We establish a correlation between LNPs structural similarity and transfection efficiency differences for the identification of pairs of molecular transfection cliffs that highlight key prediction errors.
- A large number of experiments conducted have verified the effectiveness of our model and TransLNP shows superior performance in predicting the transfection efficiency.

## ACKNOWLEDGMENTS

The authors thank the anonymous reviewers for their valuable suggestions.

## AUTHOR CONTRIBUTIONS STATEMENT

K.W, X.Y, Z.W, N.L, J.Z and L.L conceptualized and designed the study; K.W and X.Y conducted the computational analyses and drafted the initial manuscript; K.W, X.Y and Z.W contributed to the development of tables and figures. K.W, X.Y and Z.W provided significant intellectual input and were involved in the critical revision of the manuscript. L.L. supervised the entire study and ensured its adherence to the highest academic standards. All authors have read and approved the final manuscript for submission.

## Author Biographies

**Kun Wu** is a student at the Shanghai Advanced Research Institute, Chinese Academy of Sciences. His expertise lies in bioinformatics and deep learning.

**Xiulong Yang** is a researcher at the Shanghai Advanced Research Institute, Chinese Academy of Sciences. His expertise is deep learning and generative models.

**Zixu Wang** is currently a PhD student at the University of Tsukuba, Japan. His research interests include machine learning and drug discovery.

**Na Li** is the group leader of BioSAXS beamline (BL19U2) in Shanghai Synchrotron Radiation Facility. Her research interests include drug discovery, soft matter structure characterization and AI for science.

**Jialu Zhang** is a researcher at the Shanghai Advanced Research Institute, Chinese Academy of Sciences. Her expertise is bioinformatics.

**Lizhuang Liu** is a professor at the Shanghai Advanced Research Institute, Chinese Academy of Sciences. His current research interests include computer vision, bioinformatics and deep learning.
